# Nucleation control for the growth of vertically aligned GaN nanowires

**DOI:** 10.1186/1556-276X-7-373

**Published:** 2012-07-07

**Authors:** Wen-Chi Hou, Tung-Hsien Wu, Wei-Che Tang, Franklin Chau-Nan Hong

**Affiliations:** 1 Department of Chemical Engineering, Center for Micro/Nano Science and Technology, Advanced Optoelectronic Technology Center, Research Center for Energy Technology and Strategy, National Cheng Kung University, 1 University Road, Tainan, 70101, Taiwan

**Keywords:** GaN, Nanowire, Vertically aligned nanowires, Nucleation, Vapor–liquid–solid, Plasma-enhanced CVD, c-plane GaN

## Abstract

Aligned GaN nanowire arrays have high potentials for applications in future electronic and optoelectronic devices. In this study, the growth of GaN nanowire arrays with high degree of vertical alignment was attempted by plasma-enhanced CVD on the c-plane GaN substrate. We found that the lattice matching between the substrate and the nanowire is essential for the growth of vertically aligned GaN nanowires. In addition, the initial nucleation process is also found to play a key role in creating the high-quality homoepitaxy at the nanowire-substrate interface. By controlling the nucleation stage, the growth of highly aligned vertical GaN nanowire arrays can be achieved. The reasons for the observed effects are discussed.

## Background

Vertically aligned nanowires are potentially useful for the fabrication of nanowire electronic and optoelectronic devices. Both the growth direction and the crystallographic orientation of nanowires have significant effects on the efficiency, performance, and manufacturability of nanowire devices [1]. The nanowires grown via vapor–liquid–solid (VLS) or vapor-solid-solid mechanism are usually not well aligned on the substrates [2]. Compared to randomly oriented nanowires, vertically aligned nanowires have several advantages including the ability to control the crystallographic orientations of nanowires and the ability to manufacture electronic and optoelectronic devices [3]. For the vertically aligned nanowires, the uniformity of nanowire height and diameter can be more likely achieved due to the uniform mass and heat transport to each nanowire and the absence of collision and coalescence between two nanowires during the growth [4]. The devices can be also easily fabricated on the nanowire arrays with vertical alignment using the vertical electrical integration scheme, in which the efficiencies of the devices are strongly dependent on the orientation of nanowire crystals in the device [5]. Therefore, the epitaxial growth of vertically aligned nanowires has attracted a great deal of attention particularly on the growth techniques and the growth mechanism [6].

The growths of vertically aligned GaN nanowires have been demonstrated using the lattice matching or minor-mismatching substrates in several material systems, such as GaN, GaAs, InP, Si, ZnO, etc. [6-10]. Vertically aligned faceted GaN nanorods were produced by Deb et al. using a catalyst-free template approach employing a silicon dioxide mask fabricated from the porous anodic alumina [11]. George et al. reported the growth of vertically aligned GaN nanowires on the r-plane $\left(11\bar{02}\right)$ of sapphire substrate by metal-organic chemical vapor deposition (MOCVD) [12]. Li and Wang reported another route to grow ultrahigh-density and highly aligned single-crystalline GaN nanowires on sapphire by employing a submonolayer of Ni catalyst [13]. Besides, Lin et al. reported the fabrication of high-density vertically aligned GaN nanowire arrays on GaN substrate through thermal evaporation of GaN powder with the assistance of HCl gas [14]. Furthermore, Kuykendall et al. grew the vertically aligned GaN nanowires with growth orientations along $\left[11\bar{0}\right]$ and [001] on γ-LiAlO_2_ (100) and MgO (111) substrates, respectively [1]. On the other hand, the importance of slow nucleation rate on the vertical alignment of GaN nanowires has not been reported yet.

In this study, we employed the plasma-enhanced chemical vapor deposition system using Ga source and N_2_ gas reactants to synthesize GaN nanowires on the c-plane GaN film grown on sapphire by MOCVD. The homoepitaxial growth of GaN nanowires on the GaN substrate using Au catalyst allowed us to grow vertically aligned GaN nanowires. However, even for homoepitaxial growth, the growth rate at the early nucleation stage still needs to be kept low in order to grow vertically aligned GaN nanowires. The high quality of GaN nanowire crystallites was confirmed by cathodoluminescence at room temperature.

## Methods

**Figure 1:**
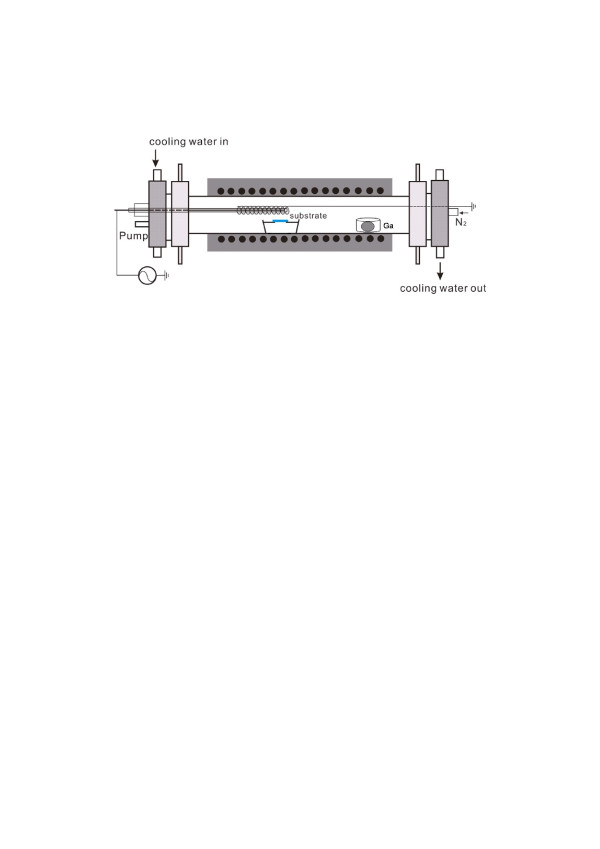
**Schematic showing the apparatus for the growth of GaN nanowires using dielectric barrier discharge (DBD).** The gallium source was located at the upstream of the reactor at a distance with respect to the substrate, and the DBD was above the substrate.

**Figure 2:**
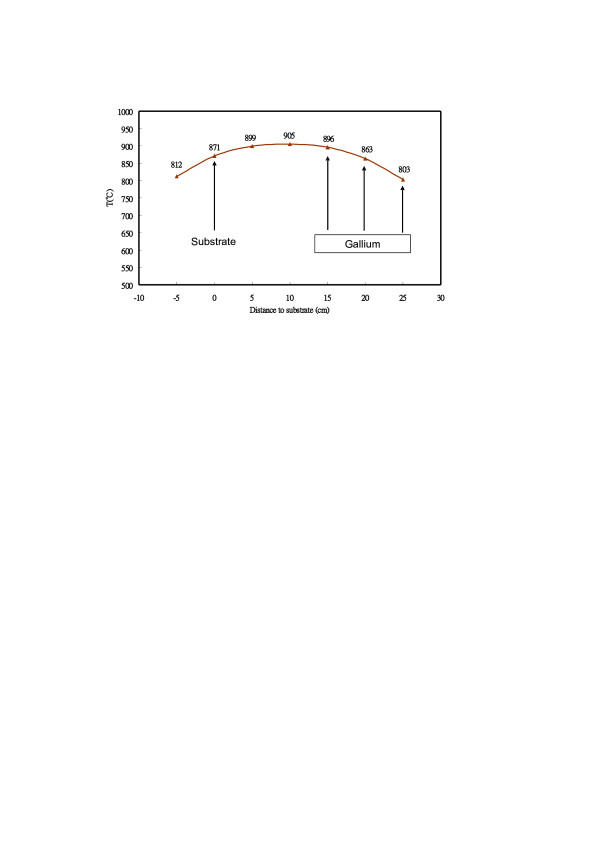
**The temperature profile of the furnace.** With its center set at 900°C, showing the temperature of Ga source at various distances to the substrate.

The experimental setup is schematically shown in Figure 1. Gallium vapor generated from heating the gallium source at the upstream of reactor flowed down to react with nitrogen radicals generated by N_2_ plasma above the substrate to grow GaN nanowires on the surface of the substrate, placed at the downstream of reactor. The position of gallium source could be adjusted in the single-zone furnace to vary the temperature of Ga source for controlling the Ga vapor pressure. We found that Ga vapor pressure was sensitive to the Ga source position in the furnace. The position of Ga source was defined by its distance with respect to the substrate, which was defined as the origin. The temperature profile of the furnace, as shown in Figure 2, was calibrated for gallium source at a distance from 15 to 25 cm. For the gallium source at a distance of 15, 20, or 25 cm to the substrate, the temperature of gallium was 896°C, 863°C or 803°C, respectively, inducing an equilibrium Ga vapor pressure of 4.2 × 10^−4^, 1.9 × 10^−4^, or 4.0 × 10^−5^ Torr. The effects of gallium vapor pressure on the GaN nanowire growth characteristics could, thus, be studied by varying the Ga source position. A 4.3-kV voltage at 9-kHz frequency was applied to the electrode above the substrate to generate a uniform dielectric barrier discharge of high-purity (99.9999%) N_2_ at a pressure of 200 Torr to produce nitrogen radicals using a power supply from Creating Nanotechnology, Inc. (Tainan City, Taiwan). The N_2_ flow rate was controlled at 100 sccm using a mass flow controller. Ga vapor, thus, reacted with nitrogen radicals in the plasma above the substrate to grow GaN nanowires. The furnace was heated at a rate of 30°C/min from room temperature to 870°C and then maintained at 870°C for 30 min to grow GaN nanowires, where the temperature at the center of the furnace was used as the furnace temperature. The N_2_ plasma for GaN growth was turned on at 500°C. The base pressure of the furnace was 1 mTorr. The c-plane GaN wafers bought from Semiconductor Wafer, Inc. (Hsinchu, Taiwan) were used as the substrates. The substrate was coated with a 3-nm-thick layer of gold (Au) and placed on top of a crucible with the growth surface facing the plasma.

The morphology of the nanowires was analyzed using scanning electron microscopy (SEM) (Philip XL-40FEG, FEI Co., Hillsboro, OR, USA). The crystal structure of nanowires was characterized by HRTEM (JEOL, JEM-2010, 200KV, JEOL Ltd., Akishima, Tokyo, Japan). Room-temperature cathodoluminescence (CL) spectra were obtained using a Gatan/Mono CL3 system (Pleasanton, CA) attached in a field-emission scanning electron microscope (JSM-7000, JEOL) with an electron beam voltage of 10 kV and a beam current of 10 nA.

## Results and discussion

**Figure 3:**
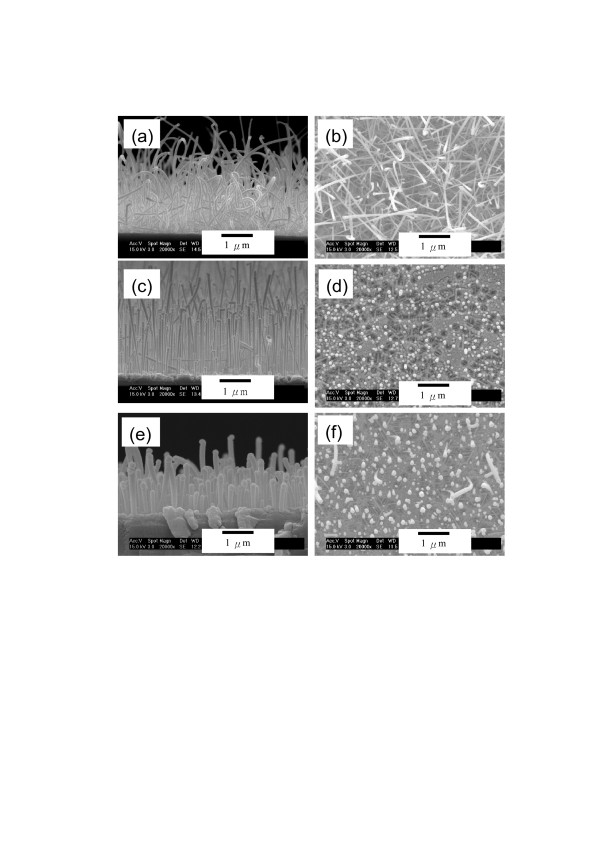
**SEM images of the GaN nanowires.** Grown on the c-plane GaN substrate with the Ga source placed at various distances to the substrate. The distances were (**a**) and (**b**) 15 cm, (**c**) and (**d**) 20 cm, and (**e**) and (**f**) 25 cm.

**Figure 4:**
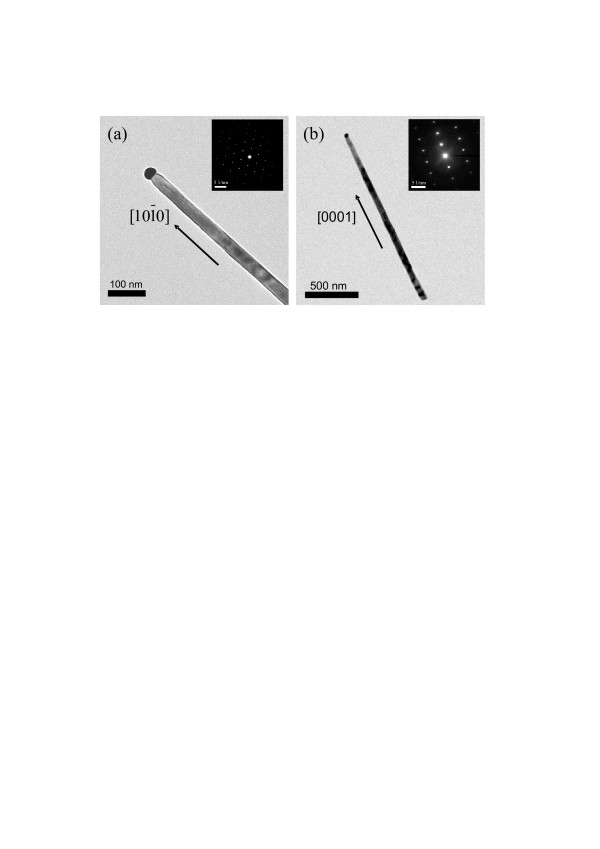
**TEM characteristics of nanowires.** Low-magnification TEM images of the nanowires grown with the Ga source at (**a**) 15- and (**b**) 20-cm distances. The insets in (a) and (b) are diffraction patterns of the nanowires in (**a**) and (**b**), respectively.

The cross-sectional and the top-view SEM images of the GaN nanowires grown with the gallium source placed at various distances to the substrate are shown in Figure 3. It should be noted that the Ga vapor pressure decreased with increasing the distance of the Ga source for the distances of 15, 20, and 25 cm. With the Ga source at a distance of 15 cm to the substrate, most of the nanowires grown were not vertically aligned to the substrate, as shown by the cross-sectional and top-view images in Figures 3a,b, respectively. In Figure 3a, the bottoms of nanowires were not vertical to the substrate indicating non-homoepitaxial growths of GaN nanowires on the GaN(0001) wafer. TEM characterizations, as shown in Figure 4a, of the GaN nanowire showed that the nanowires actually grew along $\left[101\bar{0}\right]$ orientation, confirming that no epitaxial growth was present since the nanowire growth orientation could not match the substrate orientation. As shown in the inset in Figure 4a, the selected area diffraction pattern recorded along the [0001] zone axis indicated that the nanowire axis was along $\left[101\bar{0}\right]$ orientation. Actually, $\left[101\bar{0}\right]$ is the common orientation of GaN nanowires grown on the lattice-mismatched substrates, such as c-plane sapphire or silicon [15]. As described below, the failure of homoepitaxial nanowire growth for Ga source at 15 cm was suspected to be due to the high gallium partial pressure, resulting in the high nucleation rate at the initial stage. With the Ga source at a distance of 20 cm to the substrate, the nanowires grown were found to be vertically aligned to the substrate, as shown in the cross-sectional and the top-view SEM images in Figures 3c,d, respectively. The short lengths of nanowires in the top-view image clearly indicated the good vertical alignment of GaN nanowires. The saturated vapor pressure of Ga source at 20 cm was less than one-half of that at 15 cm. The lower Ga vapor pressure at 20 cm would decrease the nucleation rate at initial stage, which might induce the growth of vertically aligned GaN nanowires. With the Ga source distance increased to 25 cm to further reduce the Ga vapor pressure, the nanowires grown were also vertically aligned, as shown by the cross-sectional and the top-view images in Figures 3e,f, respectively. Therefore, the lowering of Ga vapor pressure was shown to promote the growth of vertically aligned GaN nanowires, very likely due to the reduction of nanowire nucleation rate.

The growth rate of nanowires for Ga source at 25 cm was reduced to only one half of that for Ga source at 20 cm, as shown in Figures 3c,e, owing to the fact that the vapor pressure of Ga at 25 cm was only one fourth of that at 20 cm. However, the average diameter of the nanowires for Ga at 25 cm was about 150 nm, which was around twice as large as those for Ga at 15 and 20 cm. In the VLS growth of nanowires, the extremely thin Au film (3-nm thick) would sinter into Au nanoparticles before the nucleation of GaN nanowires due to the Ostwald ripening mechanism, and the diameter of nanowires was dependent on the size of the catalyst nanoparticles. During heating of the furnace from room temperature, the Ga source and the substrate were both gradually heated up. When the Ga source was heated to a temperature providing enough concentration of Ga vapor, the nucleation of GaN nanowires would start to occur through the reaction of Ga vapor with nitrogen plasma. The substrate for the Ga source at 25 cm needed to be heated up to a temperature much higher and longer than those at 15 and 20 cm to allow the Ga source to reach the same temperature as those at 15 and 20 cm for providing the same Ga vapor pressure to initiate the GaN nanowire nucleation. For the Ga source at 25 cm, the higher substrate temperature and longer heating period to initiate nucleation resulted in a higher degree of sintering forming Au catalyst nanoparticles of larger sizes, which resulted in the growth of GaN nanowires with a larger diameter.

**Figure 5:**
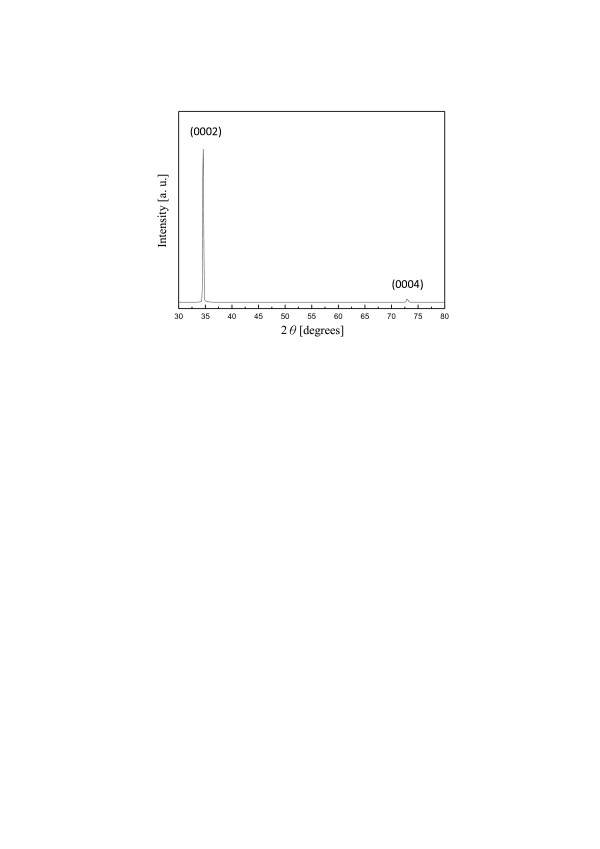
**XRD pattern of vertically aligned GaN nanowires.** Grown with the Ga source at a distance of 20 cm.

The GaN nanowires grown with Ga source at 20 cm were also characterized by TEM. In Figure 4b, the catalyst at the tip of nanowire indicated that the growth of nanowire proceeded by VLS mechanism. In the insect of Figure 4b, the diffraction pattern recorded along the $\left[011\bar{0}\right]$ zone axis indicated that the nanowire axis was along [0001] orientation. It suggested that the vertical GaN nanowires were grown on GaN(0001) substrate through the homoepitaxial growth of GaN nanowires. Figure 5 shows the XRD results of the sample in Figure 3c. Two XRD peaks of (0002) and (0004) attributed from the wurtzite GaN were detected, indicating the vertical alignment of GaN nanowires. Since the growth of GaN nanowires was vertical only under low Ga vapor pressure, the homoepitaxial growth of GaN nanowires would most likely require a slow growth process during the nucleation of nanowires at the catalyst-GaN substrate interface. In other words, the Ga and N atoms diffusing out of the catalyst need enough time to migrate to the lowest energy sites forming homoepitaxial interface on GaN(0001).

**Figure 6:**
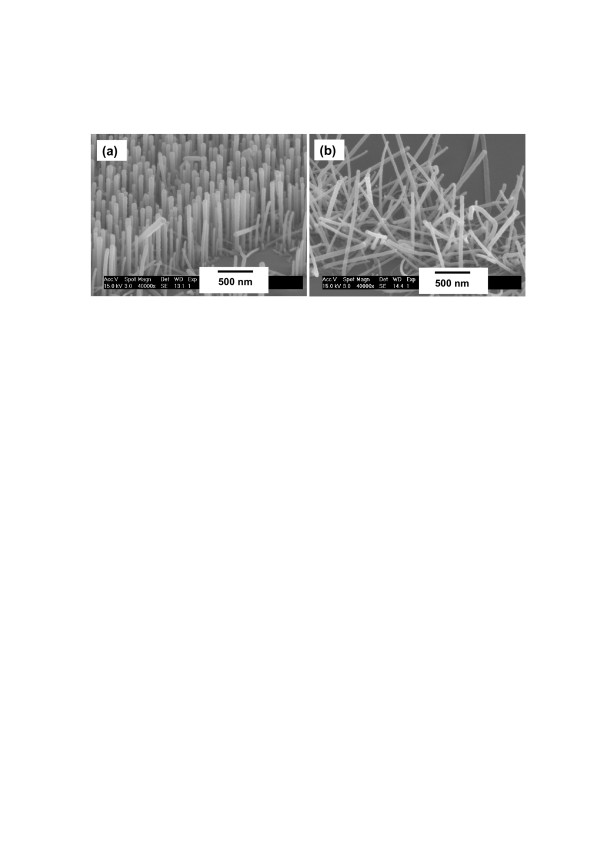
**Tilted SEM images of GaN nanowires.** Grown with the Ga source at a distance of 20 cm to the substrate on the (**a**) c-plane GaN and (**b**) c-plane sapphire substrates.

To understand the role of substrate in the growth of vertically aligned nanowires, we also compared the nanowire growths on the lattice-matched and lattice-mismatched substrates using the optimized conditions by placing the Ga source at 20 cm to induce a slow growth at the nucleation stage. With the Ga source at 20 cm, the GaN nanowires were grown on the c-plane GaN and c-plane sapphire substrates, respectively, as shown in Figures 6a,b. The calculated mismatch between the c-plane (0001) GaN and the c-plane (0001) sapphire is larger than 30%. However, the actual mismatch is smaller (approximately 16%) because the small cell of Al atoms on the basal sapphire plane is oriented 30° away from the larger sapphire unit cell [16]. Figure 6 clearly shows that the nanowires grown on the sapphire substrate were not vertically aligned, but those on the GaN substrate were well aligned vertically. Therefore, the lattice matching between the nanowires and the substrate was essential for the growth of vertically aligned nanowires, besides the requirement of a low growth rate at the nanowire nucleation stage.

**Figure 7:**
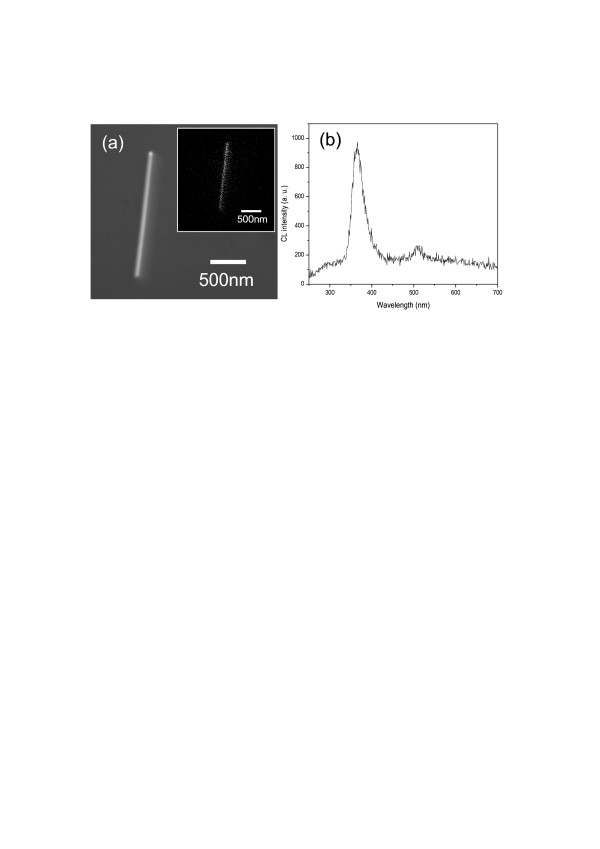
**CL spectra of a GaN nanowire.** Grown with the Ga source at a distance of 20 cm. (**a**) SEM image, along with the 366-nm monochromatic luminescence image in the inset, and (**b**) CL spectra.

A single GaN nanowire grown with the Ga source at a distance of 20 cm from the substrate was analyzed by CL spectroscopy. As shown in Figure 7a, the nanowire was transferred from the native GaN substrate to a blank silicon substrate using a PDMS stamp. The room-temperature CL spectra shown in Figure 7b revealed a strong band-to-band emission at around 366 nm without any defect luminescence. The small peak at 510 nm was contributed from the interface of silicon substrate and the native silicon oxide [17]. The inset in Figure 7a shows the monochromatic CL image at 366 nm for the nanowire in the main picture. The major luminescence at 366 nm was demonstrated to be contributed uniformly from the whole nanowire. Also, the negligible defect luminescence in Figure 7b illustrates that the defect concentration in the GaN nanowires, grown with the Ga source at 20 cm, was extremely low.

## Conclusion

A route to highly vertically aligned GaN nanowires on the c-plane GaN substrate is reported in this study. We have shown that the degree of vertical alignment can be improved via controlling the gallium partial pressure during the nucleation of nanowires on the substrate. First, the lattice-matched substrate is an essential requirement for the growth of vertically aligned GaN nanowires. In addition, the nucleation stage plays a key role in the vertical alignment of GaN nanowires by creating homoepitaxial interfaces between the nanowires and the substrate. As a result, a slow growth rate at the nucleation stage is required for the homoepitaxial growth of nanowires. The CL analysis has further shown that the DBD-type nitrogen plasma employed in this study can supply sufficient active nitrogen species to react with Ga vapor forming high quality GaN nanowire crystallites.

## Competing interests

The authors declare that they have no competing interests.

## Authors’ contributions

WH wrote the manuscript and carried out the synthesis and SEM measurements. TW carried out the XRD and CL measurements. WT carried out the TEM measurements. FH conceived of the study, participated in its design and coordination, and drafted the manuscript. All authors read and approved the final manuscript.
